# Markers of Skeletal Muscle Mitochondrial Function and Lipid Accumulation Are Moderately Associated with the Homeostasis Model Assessment Index of Insulin Resistance in Obese Men

**DOI:** 10.1371/journal.pone.0066322

**Published:** 2013-06-12

**Authors:** Imtiaz A. Samjoo, Adeel Safdar, Mazen J. Hamadeh, Alexander W. Glover, Nicholas J. Mocellin, Jose Santana, Jonathan P. Little, Gregory R. Steinberg, Sandeep Raha, Mark A. Tarnopolsky

**Affiliations:** 1 Department of Pediatrics, McMaster University, Hamilton, Ontario, Canada; 2 Department of Medicine, McMaster University, Hamilton, Ontario, Canada; 3 Cardiovascular Institute, Beth Israel Deaconess Medical Centre, Harvard Medical School, Boston, Massachusetts, United States of America; 4 School of Kinesiology and Health Science, Muscle Health Research Centre, York University, Toronto, Ontario, Canada; 5 School of Health and Exercise Sciences, University of British Columbia Okanagan, Kelowna, British Columbia, Canada; Pennington Biomed Research Center, United States of America

## Abstract

Lower skeletal muscle mitochondrial oxidative phosphorylation capacity (OXPHOS) and intramyocellular lipid (IMCL) accumulation have been implicated in the etiology of insulin resistance (IR) in obesity. The purpose of this study was to examine the impact of endurance exercise on biochemical and morphological measures of IMCL and mitochondrial content, and their relationship to IR in obese individuals. We examined mitochondrial content (subunit protein abundance and maximal activity of electron transport chain enzymes), IMCL/mitochondrial morphology in both subsarcolemmal (SS) and intermyofibrillar (IMF) regions by transmission electron microscopy, and intracellular lipid metabolites (diacylglycerol and ceramide) in *vastus lateralis* biopsies, as well as, the homeostasis model assessment index of IR (HOMA-IR) prior to and following twelve weeks of an endurance exercise regimen in healthy age- and physical activity-matched lean and obese men. Obese men did not show evidence of mitochondrial OXPHOS dysfunction, disproportionate IMCL content in sub-cellular regions, or diacylglycerol/ceramide accretion despite marked IR *vs.* lean controls. Endurance exercise increased OXPHOS and mitochondrial size and density, but not number of individual mitochondrial fragments, with moderate improvements in HOMA-IR. Exercise reduced SS IMCL content (size, number and density), increased IMF IMCL content, while increasing IMCL/mitochondrial juxtaposition in both regions. HOMA-IR was inversely associated with SS (r = −0.34; *P* = 0.051) and IMF mitochondrial density (r = −0.29; *P* = 0.096), IMF IMCL/mitochondrial juxtaposition (r = −0.30; *P* = 0.086), and COXII (r = −0.32; *P* = 0.095) and COXIV protein abundance (r = −0.35; *P* = 0.052); while positively associated with SS IMCL size (r = 0.28; *P* = 0.119) and SS IMCL density (r = 0.25; *P* = 0.152). Our findings suggest that once physical activity and cardiorespiratory fitness have been controlled for, skeletal muscle mitochondrial and IMCL profile in obesity may only partially contribute to the development of IR.

## Introduction

Obesity is a major risk factor for the development of insulin resistance and many chronic diseases, such as type 2 diabetes mellitus (T2D) [1]. Although the causal factor mediating insulin resistance in obesity remains elusive, defects in skeletal muscle mitochondrial function [2], [3], [4], [5], [6], [7], [8] including aberrant mitochondrial morphology [2], [9] and reduced expression of genes responsible for oxidative metabolism [10], [11] leading to accumulation of intramyocellular lipid (IMCL) have been proposed as mechanisms mediating the disease process. As such, several studies have shown an inverse association between skeletal muscle IMCL content and whole-body insulin sensitivity in obese individuals [12], [13] and those with T2D [14], [15], [16]. Similarly, intracellular fatty acid intermediates, such as diacylglycerol (DAG) and/or ceramides, possibly produced due to impaired mitochondrial metabolism, have been shown to interfere with insulin signaling [17], [18], [19], [20]. These findings have led to the hypothesis that reduced mitochondrial content and/or oxidative capacity in skeletal muscle contributes to insulin resistance - a hallmark of obesity and T2D [8], [21], [22].

Skeletal muscle is composed of two distinct mitochondrial subpopulations, i.e., subsarcolemmal (SS) and intermyofibrillar (IMF) mitochondria [23]. Ritov *et al.* reported that electron transport chain (ETC) activity of SS mitochondria was lower in obese individuals with or without T2D *vs.* lean controls [3]. A more recent report indicated that IMF mitochondria content was lower in subjects with T2D but SS mitochondrial content was similar compared with insulin-sensitive subjects [24]. Much like mitochondrial subpopulations, skeletal muscle is composed of two distinct IMCL pools, which also reside in SS and IMF regions. Despite a direct association between aberrant IMCL accumulation and increased insulin resistance [13], [25], [26], there is a paucity of data specifically examining the potential alteration in SS and IMF IMCL pools in obesity or T2D [27]. Hence, it remains to be elucidated if differential alterations in mitochondrial and IMCL subpopulations are etiologically linked to the development of insulin resistance/T2D.

It is important to note that in the aforementioned studies [3], [24] physical fitness was not strictly controlled which is intimately associated with skeletal muscle mitochondrial dysfunction [28]. It is becoming extremely important to define and match subjects (lean *vs.* obese *vs.* T2D) based on their physical activity and fitness levels to prevent the confounding effect of sedentary lifestyle on muscle mitochondrial metabolism and IMCL content. Much like obese and T2D subjects, endurance-trained athletes also exhibit higher IMCL content, coupled with an increased abundance of mitochondria and heightened insulin sensitivity [15], [26], [29]. This paradox points to physical activity as a major determinant of the IMCL/mitochondrial relationship in human skeletal muscle. This paradox has also challenged the concept that the accumulation of IMCL within skeletal muscle is invariably linked to insulin resistance.

Taken together, it is evident that the nature of mitochondrial functional pathology and the causal axis between mitochondrial dysfunction, aberrant IMCL accumulation, and development of insulin resistance/T2D remains elusive. Furthermore, the impact of endurance exercise on the compartmentalization of IMCL droplets and mitochondria is poorly understood. The current study aimed to clarify this association by examining several biochemical measures of mitochondrial components which are used as biomarkers of mitochondrial content as well as morphological measures of mitochondrial content in skeletal muscle of obese men and their age- and physical activity-matched lean counterparts. We also examined the influence of moderate-intensity endurance exercise training on skeletal muscle adaptations in both lean and obese subjects.

## Materials and Methods

### Subjects

Men were recruited through local advertisements and underwent a telephone and an in-person interview to assess eligibility. The Research Ethics Board of McMaster University approved the experimental protocol (REB project #: 05–053), and subjects provided written informed consent prior to participation in accordance with the *Declaration of Helsinki*. Inclusion criteria included age from 20–55 years and body mass index (BMI) of 18.5–24.9 kg/m^2^ for lean and ≥30.0 kg/m^2^ for obese individuals with a self-reported stable body weight during the previous 6 months. Individuals who had evidence of T2D, hypertension (>140/90 mmHg), and/or an abnormal exercise stress test, smoked, had orthopedic contraindications to physical activity, or used lipid-lowering, glucose-lowering, antihypertensive, antidepressant or weight-loss medications, or consumed more than two alcoholic beverages per day were excluded. Participants were only involved in routine activities of daily living (walking, gardening, etc.) and not engaged in regular structured or individualized aerobic or strength training programs or athletics. Twenty-four men enrolled in the study, and experimental groups were matched for age and cardiorespiratory fitness (VO_2peak_/kg FFM/min) when corrected for fat-free mass FFM (Table 1). Six men (3 in the lean group, 3 in the obese group) did not complete the study because of inability/unwillingness to comply with protocol or due to personal or work-related conflicts.

**Table 1:** Participant characteristics.

	Lean		Obese	
	Pre-training	Post-training	Pre-training	Post-training
*n*	9		9	
Age (yr)	38±3		39±3	
Height (cm)	179±3		180±3	
Body weight (kg)	75.5±3.4	75.2±3.2	108.4±6.1^A^	107.1±6.5^B^
BMI (kg.m^−2^)	23.6±0.5	23.5±0.5	33.6±1.6^A^	33.1±1.7^B^
Waist circumference (cm)	86.2±1.4	82.5±1.7^C^	111.8±3.7^A^	108.6±4.2B,C
Fat mass (kg)	14.9±1.9	14.4±2.1	35.7±3.3^A^	35.2±3.5^B^
Fat-free mass (kg)	57.3±2.6	57.9±2.5	68.8±2.7^A^	68.2±2.9^B^
Body fat (%)	20.5±2.3	19.7±2.5	33.7±1.4^A^	33.5±1.5^B^
Aerobic capacity (mL O_2_.kg^−1^ fat-free mass.min^−1^)	46.9±2.1	55.5±2.4^C^	44.6±2.1	51.1±2.0^C^

Data are presented as means ± SEM.

A Obese group data significantly different from lean group data, *P*≤0.01.

B Obese group data significantly different from lean group data, *P*≤0.02.

C Post-training significantly different from pre-training (main effect), *P*≤0.001.

### Protocol

All subjects underwent a 12-week endurance exercise training protocol on a stationary cycle ergometer (Monarck, Cardio Care 827 E), as previously described [30]. Briefly, the protocol commenced with two 30-min biking sessions at 50% VO_2peak_ per week in the first week and increased to three 60-min biking session at 70% VO_2peak_ per week by the final week of training. To ensure that subjects were cycling at the appropriate intensity, heart rate (a proxy for VO_2peak_) was used to monitor training intensity based on heart rate measurements obtained during the VO_2peak_ test.

### Metabolic Assessments

Prior to and following the intervention (48-h after the last training session), all participants underwent evaluation of insulin resistance, body composition, physical fitness, and had a muscle biopsy. After an overnight fast, the glycemic response to a 75-g oral glucose load (300 mL) was determined. Blood samples were collected before and 30, 60, 90 and 120 min during the oral glucose tolerance test (OGTT). For estimation of whole-body insulin resistance from data obtained during the OGTT, the homeostasis model assessment index of insulin resistance (HOMA-IR) [31] was calculated according to the following equation: HOMA-IR = I_0_×G_0_/22.5; where I_0_ is the fasting insulin concentration (in µU/mL), G_0_ is the fasting glucose concentration (in mM). The HOMA-IR has been validated against the euglycemic-hyperinsulinemic clamp, with correlations ranging from r = −0.725, *P*<0.0001 [32] to Rs = 0.88, *P*<0.0001 [31]. Fat mass, FFM, and body fat percentage were assessed by dual energy X-ray absorptiometry (GE Lunar, Prodigy, Madison, WI). A symptom-limited maximal oxygen consumption test (VO_2peak_) was determined on an electronically braked cycle ergometer and a computerized open-circuit gas collection system (Moxus Modulator VO_2_ system with O_2_ analyzer S-3A/I and CO_2_ analyzer CD-3A, AEI Technologies Inc., Pittsburgh, PA). Subjects cycled (Excalibur Sport, Lode, Groningen, Netherlands) at 50 W for 1 min, thereafter increasing in increments of 25 W/min. VO_2peak_ was established when O_2_ consumption values reached a plateau or was the highest value during the incremental ergometer protocol, pedal revolutions could not be maintained over 60 rpm despite vigorous encouragement, and the respiratory exchange ratio was more than 1.12. Subjects were monitored using a 12-lead ECG to rule out any cardiovascular abnormalities.

### Blood Sample Analysis

Blood samples were taken from the antecubital vein after an overnight fast, collected in heparinized vials, placed on ice, centrifuged at 1750 *g* for 10 min, and stored at −80°C until subsequent analysis. Serum free-fatty acid (FFA) concentration was determined using a commercially available ELISA kit (NEFA kit, Wako Diagnostics, Richmond, VA). Plasma glucose concentration was determined using an automated glucose analyzer (2300 STAT plus, YSI, UK). Plasma insulin concentration was determined using a commercially available ELISA kit (INS kit, BioSource, Belgium, EU).

### Muscle Biopsies

Samples of *vastus lateralis* were obtained after an overnight fast, as previously described [29]. Biopsies were taken from the same leg prior to and following the intervention with 3–5 cm between the incision sites. Approximately 150 mg muscle tissue was obtained each time and immediately dissected of any adipose and connective tissue. A portion was saved for transmission electron microscopy (TEM) analysis and the remainders immediately flash frozen in liquid nitrogen. Samples were stored at −80°C for subsequent biochemical and molecular analysis.

### Transmission Electron Microscopy

TEM was used to determine IMCL and mitochondrial characteristics, as previously described [29]. Samples were viewed at 6,500× using a JEOL 1200EX transmission electron microscope. Sixteen micrographs were acquired from 8 randomly sampled longitudinal sections of muscle fibers (2 micrographs/fiber) from each individual muscle - one micrograph acquired near the cell surface representing the SS region and the other acquired of parallel bundles of myofibrils representing the IMF region. Lipid droplets and mitochondrial fragments were circled and converted to actual size using a calibration grid. For each set of 16 images, mean IMCL or mitochondrial size (µm^2^), total number of IMCL droplets or mitochondria per square micrometer of tissue (#/µm^2^), percentage IMCL or mitochondrial area density (i.e., the fraction of cell area occupied by IMCL or mitochondria), and the percentage of IMCL in contact with mitochondria were calculated in the IMF and SS compartments by digital imaging software (Image Pro Plus, ver. 4.0; Media Cybernetics, Silver Springs, MD), as previously described [29]. The reference for SS space quantification was the cytoplasmic space between the sarcolemma and the first layer of myofibrils.

### Homogenization and Immunoblotting

Total protein was extracted from frozen biopsy samples, as previously described [29]. The Lowry assay was used to quantify the total protein content [33]. Proteins were resolved on either 7.5, 10 or 12.5% SDS-PAGE gels, transferred onto Hybond® ECL nitrocellulose membranes (Amersham), and immunoblotted using the following commercially available primary antibodies: anti-COX subunit II (cytochrome *c* oxidase - subunit II, MS405) and anti-COX subunit IV (cytochrome *c* oxidase - subunit IV, MS408) were purchased from MitoSciences; anti-GLUT4 from Chemicon (ab1346); anti-phospho-Akt (Ser^473^, 4060) and anti-PGC-1α (peroxisome proliferator-activated receptor-γ coactivator-1α, 4187) from Cell Signaling Technology. The anti-CS (citrate synthase) antibody was a generous gift by Dr. Brian Robinson (The Hospital for Sick Children, Toronto, ON). Anti-β-actin (612657, BD Biosciences) was used as a loading control. Membranes were then incubated with the appropriate anti-mouse or anti-rabbit horseradish peroxidase-conjugated secondary antibody and visualized by enhanced chemiluminescence detection reagent (Amersham). Relative intensities of the protein bands were digitally quantified using ImageJ Version 1.37 statistical analysis software.

### Diacylglycerol and Ceramide

Muscle DAG and ceramide content was determined as previously described [34]. Briefly, lipids were extracted from freeze-dried muscle, and DAG kinase and [^32^P]ATP were added to samples preincubated with cardiolipin/oxtylglucoside and allowed to react for 2 h. The reaction was stopped and samples were spotted onto thin-layer chromatography plates and developed. Bands representing ^32^P-labelled phosphatidic acid and ceramide-1-phosphate were dried, scraped from the plate and counted using a liquid scintillation analyzer (Tri-Carb, 2500TR).

### Enzyme Activity

Muscle lysate CS (EC 2.3.3.1), complex IV (COX, EC 1.9.3.1), and β-oxidation (short-chain β-hydroxyacyl-CoA dehydrogenase, SCHAD) activity was determined, as previously described [29]. All samples were analyzed in duplicate on a UV spectrophotometer (Cary 300 Bio UV-Visible spectrophotometer, Varian, Palo Alto, CA) and expressed as nmol.min^−1^.mg protein^−1^.

### Total DNA Isolation

Total DNA was isolated from ∼15 mg of skeletal muscle using the Qiagen total DNA isolation kit (Qiagen, Mississauga, ON) according to the manufacturer’s instructions. DNA samples were treated with RNase (Fermentas, Mississauga, ON) to remove RNA contamination. DNA concentration and quality was assessed using Nanodrop 2000 (Thermo Scientific, Wilmington, DE).

### Mitochondrial DNA Content

Mitochondrial DNA (mtDNA) copy number, relative to the diploid chromosomal DNA content was quantitatively analyzed in skeletal muscle using ABI 7300 real-time PCR (Applied Biosystems, CA). Primers were designed around ND1 (forward primer, L3485–3504; reverse primer, H3532–3553) and ND4 (forward primer, L12087–12109; reverse primer, H12140–12170) regions of the mitochondrial genome. Nuclear β-globin gene was used as a housekeeping gene.

### Statistical Analysis

When analyzing differences between lean and obese individuals, statistical analyses were completed using unpaired Student’s *t*-tests for independent samples (Statistica, Version 5.0, Statsoft, Tulsa, OK) with adiposity (lean, obese) being the experimental condition. A two-way repeated measures ANOVA (Statistica, Version 5.0, Statsoft, Tulsa, OK) with adiposity (lean, obese) and training (pre, post) being the experimental conditions was completed when analyzing the effect of the endurance exercise training program. When statistical significance was achieved, a Tukey’s HSD post-hoc test was used to identify individual differences. Pearson correlation analyses were performed using GraphPad Prism (Version 4, GraphPad Software, San Diego, CA). Statistical significance was established at *P*≤0.05. Data are presented as means ± SEM.

## Results

### Lean and Obese Subject Baseline Characterization

#### Subject characteristics

Lean and obese men were group matched for age and aerobic capacity. A comprehensive anthropometrical description of the participants is provided in Table 1 and Table 2. Body weight, BMI, waist circumference, fat mass, FFM, and body fat percentage were markedly higher in obese men *vs.* lean men (Table 1). Fasting serum triglyceride and FFA levels were significantly elevated, whereas fasting serum HDL cholesterol concentrations were significantly lower in obese men *vs.* lean men (Table 2).

**Table 2:** Metabolic characteristics.

	Lean		Obese	
	Pre-training	Post-training	Pre-training	Post-training
Total cholesterol (mM)	4.8±0.4	4.6±0.3	5.3±0.4	5.3±0.4
Triglyceride (mM)	0.9±0.1	0.8±0.1	1.5±0.2^A^	1.5±0.2^B^
HDL cholesterol (mM)	1.5±0.1	1.4±0.1	1.2±0.1^A^	1.2±0.1
LDL cholesterol (mM)	2.8±0.3	2.8±0.3	3.4±0.3	3.4±0.3
FFA (mM)	0.3±0.1	0.5±0.1^C^	0.5±0.1^A^	0.6±0.1^C^
FPG (mM)	5.4±0.1	5.5±0.1	5.7±0.2	5.7±0.2
FPI (µU.mL^−1^)	8.1±0.9	7.6±0.9	12.1±1.4^A^	10.2±1.1
2-h PG (mM)	4.9±0.2	4.4±0.3^C^	7.1±0.7^A^	5.4±0.6^C^
2-h PI (µU.mL^−1^)	25.1±6.6	26.5±4.9	74.3±16.9^A^	39.1±5.2

Data are presented as means ± SEM.

A Obese group data significantly different from lean group data, *P*≤0.04.

B Obese group data significantly different from lean group data, *P*≤0.01.

C Post-training significantly different from pre-training (main effect), *P*≤0.03.

2-h PG, 2-h plasma glucose; 2-h PI, 2-h plasma insulin; FFA, free fatty acid; FPG, fasting plasma glucose; FPI, fasting plasma insulin.

#### Oral glucose tolerance test and insulin resistance

**Figure 1:**
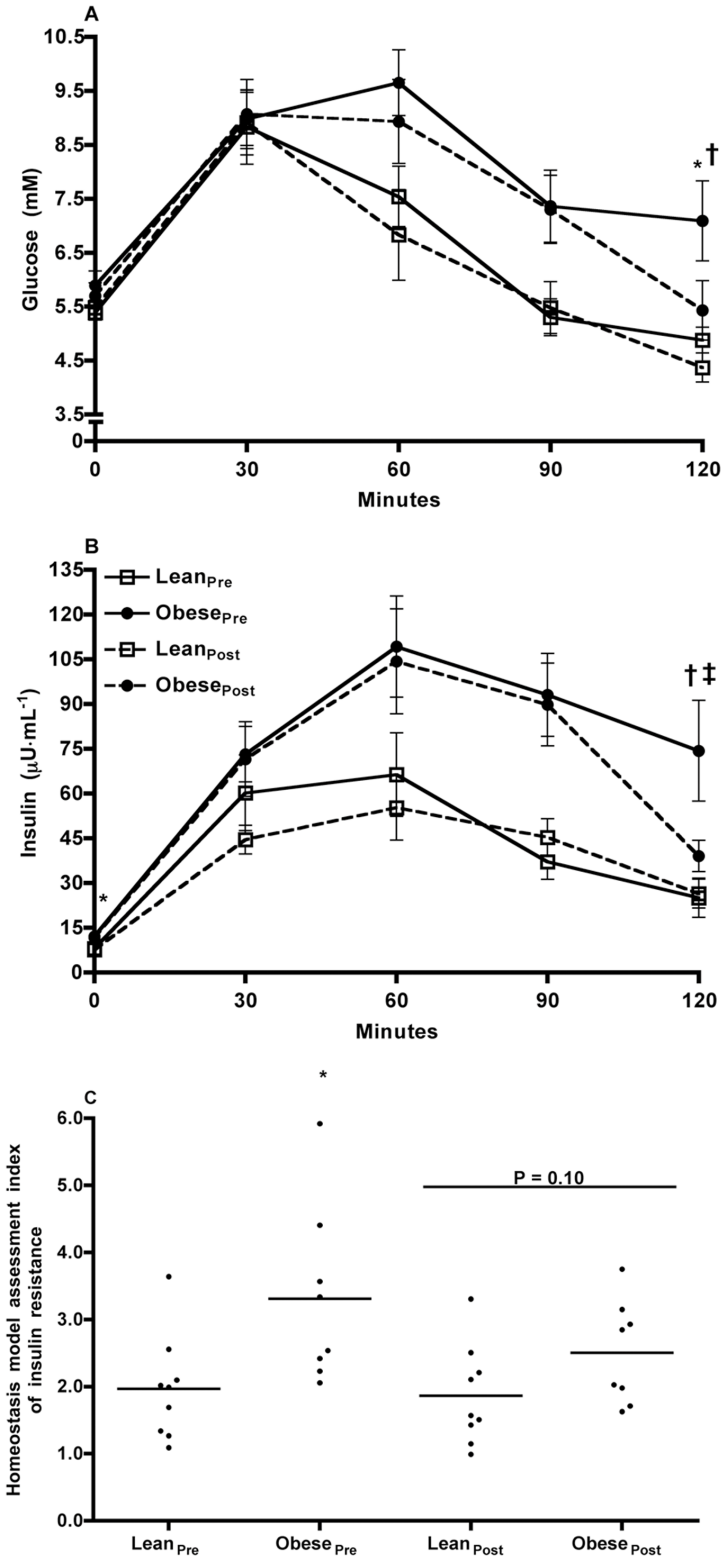
Results of the oral glucose tolerance test. Mean plasma concentrations of glucose (**A**) and insulin (**B**) during a 75-g oral glucose tolerance test, and (**C**) the homeostasis model assessment index of insulin resistance (HOMA-IR) in lean (*n* = 9) and obese (*n* = 9) men prior to and following 12-wk endurance training. (**A**) *P* = 0.04 and *P* = 0.28 for the comparison of the areas under the curve for glucose (AUC_glucose_) of lean and obese men, pre- and post-training, respectively. 2-hr plasma glucose concentration: *P≤0.01 lean *vs.* obese pre-training; † *P*≤0.01 pre- *vs.* post-training (main effect). (**B**) *P* = 0.02 and *P* = 0.02 for the comparison of the areas under the curve for insulin (AUC_insulin_) of lean and obese men, pre- and post-training, respectively. Fasting plasma insulin concentration: *P≤0.03 lean *vs.* obese pre-training; 2-hr plasma insulin concentration: ^†^ *P*≤0.02 lean *vs.* obese pre-training; ^‡^P = 0.07 pre- *vs.* post-training (main effect). (**C**) HOMA-IR was 68% higher in the obese *vs.* lean men pre-training and decreased by 17% post-training. *P≤0.02 lean *vs.* obese pre-training; *P* = 0.10 pre- *vs.* post-training (main effect).

2-h plasma glucose levels as well as fasting and 2-h plasma insulin levels were significantly higher in obese men *vs.* lean men (Table 2). Plasma concentrations of glucose (AUC_glucose_; Figure 1A) and insulin (AUC_insulin_; Figure 1B) during the OGTT were significantly higher in obese men *vs.* lean men. Assessment of insulin resistance with the HOMA-IR showed 68% greater insulin resistance in obese men *vs.* lean men (3.31±0.47 *vs.* 1.97±0.26, *P*≤0.02; Figure 1C).

#### Biomarkers of mitochondrial content

**Figure 2:**
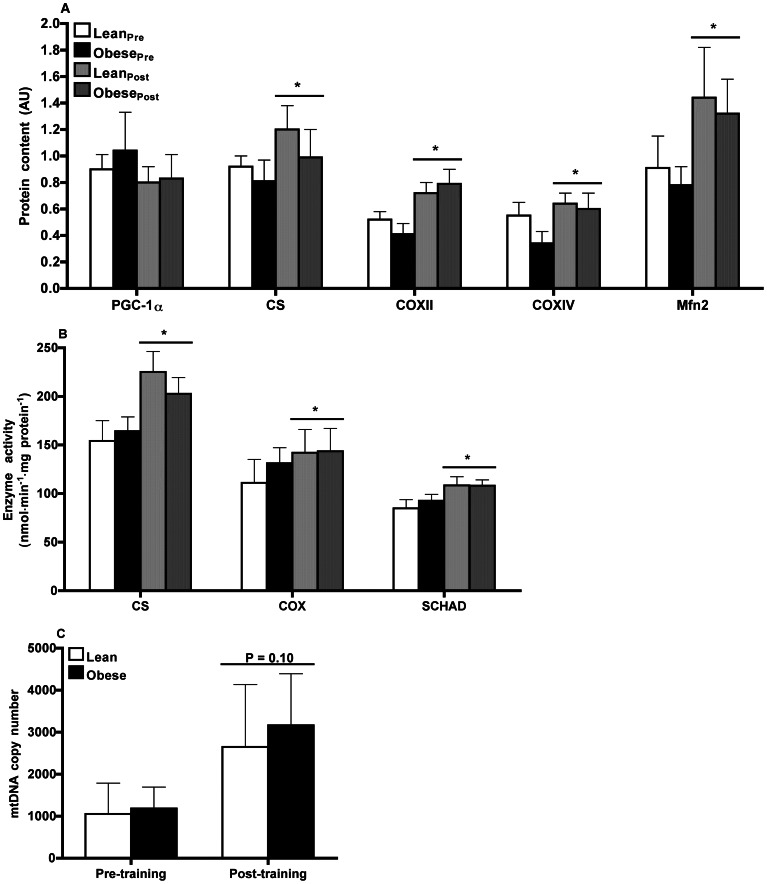
Markers of mitochondrial function. (**A**) Mitochondrial protein content assessed by Western blot and (**B**) mitochondrial maximal enzyme activity in skeletal muscle of lean (*n* = 9) and obese (*n* = 9) men prior to and following 12-wk endurance training. (**A**) PGC-1α, peroxisome proliferator-activated receptor-γ coactivator-1α; CS, citrate synthase; COX, cytochrome *c* oxidase - subunits II and IV. Results were normalized to β-actin protein content. *P≤0.04 pre- *vs.* post-training (main effect). (**B**) CS, citrate synthase; COX, cytochrome *c* oxidase; SCHAD, short-chain β-hydroxyacyl-CoA dehydrogenase. *P≤0.03 pre- *vs.* post-training (main effect). (**C**) Mitochondrial DNA (mtDNA) copy number determined by real-time quantitative PCR using a TaqMan probe against NADH dehydrogenase 4 (ND4) and β-globin. mtDNA copy number was calculated as the ratio of ND4 to β-globin in skeletal muscle of lean (*n* = 3) and obese (*n* = 5) men prior to and following 12-wk endurance training. *P = 0.10 pre- *vs.* post-training (main effect).

The transcriptional co-activator PGC-1α is widely regarded as the master regulator of mitochondrial biogenesis [35], [36]. We found no difference in whole muscle protein content of PGC-1α between lean and obese men (Figure 2A). We further analyzed the expression of several key nuclear- and mtDNA encoded proteins that are involved in mitochondrial energy metabolism. The protein content of CS (marker of mitochondrial abundance), COX subunit II (COXII; mtDNA-encoded) and subunit IV (COXIV; nuclear DNA encoded), as well as the maximal activities of CS, COX, and SCHAD (marker of mitochondrial β-oxidation) were similar in both lean and obese men (Figure 2, A and B). We also measured mtDNA copy number in skeletal muscle and observed no between-group differences (Figure 2C).

#### Intramyocellular lipid and mitochondrial content

**Figure 3:**
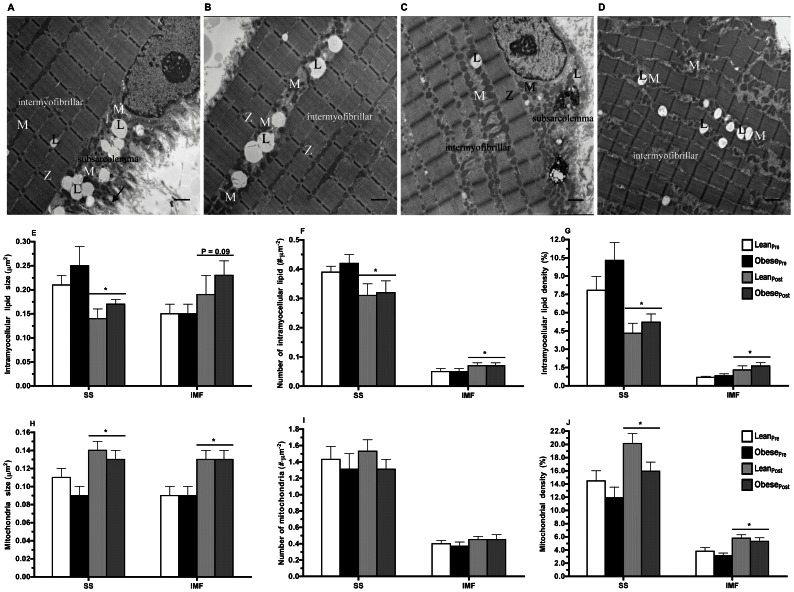
Transmission electron microscopy assessment of intramyocellular lipid and mitochondrial content. Micrographs of a skeletal muscle cell illustrating subsarcolemmal (**A**) and intermyofibrillar (**B**) intramyocellular lipid (IMCL) and mitochondria prior to (**A**,**B**) and following 12-wk endurance training (**C**,**D**). Subsarcolemmal (SS) IMCL and mitochondria are located between the sarcolemma and the most superficial myofibrils. The intermyofibrillar (IMF) IMCL and mitochondria are located between parallel bundles of myofibrils. The micrographs (X6,500 magnification, scale bar: 1 µm) were obtained from a biopsy of the *vastus lateralis* muscle from an obese participant. L, intramyocellular lipid droplet; M, mitochondria, Z, Z-line. IMCL size (**E**), number (**F**), and density (**G**) in SS and IMF regions of skeletal muscle of lean (*n* = 9) and obese (*n* = 9) men prior to and following 12-wk endurance training. *P≤0.05 pre- *vs.* post-training (main effect). Mitochondria size (**H**), number (**I**), and density (**J**) in SS and IMF regions of skeletal muscle of lean (*n* = 9) and obese (*n* = 9) men prior to and following 12-wk endurance training. *P≤0.01 pre- *vs.* post-training (main effect).

**Figure 4:**
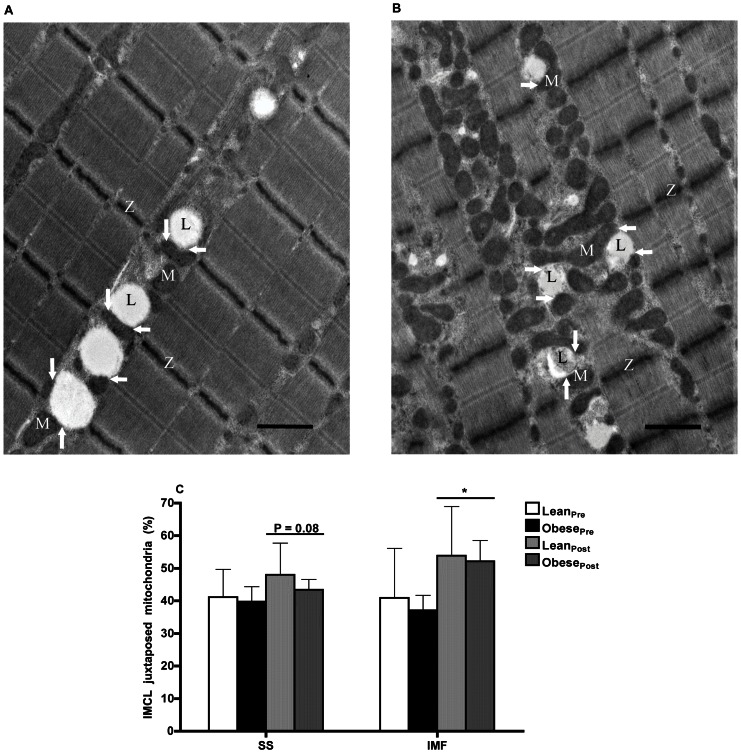
Transmission electron microscopy assessment of intramyocellular lipid and mitochondrial proximity. Representative electron micrographs of a skeletal muscle cell illustrating subsarcolemmal (**A**) and intermyofibrillar (**B**) intramyocellular lipid (IMCL) juxtaposed with mitochondria prior to (**A**,**B**) and following 12-wk endurance training (**C**,**D**). The micrographs (X6,500 magnification, scale bar: 1 µm) were obtained from a biopsy of the *vastus lateralis* muscle from an obese participant. Graph represents the proportion of IMCL juxtaposed with mitochondrial (i.e., the proportion of IMCL in contact with mitochondria) in subsarcolemmal and intermyofibrillar regions of skeletal muscle of lean (*n* = 9) and obese (*n* = 9) men prior to and following 12-wk endurance training. *P≤0.02 pre- *vs*. post-training (main effect).

IMCL and mitochondria are heterogeneously distributed in myofibers. Two sub-cellular fractions of IMCL and mitochondria were characterized based on their location in both the SS and IMF regions. Electron micrographs illustrating these subpopulations are shown in Figure 3A–D. A summary of both IMCL and mitochondrial morphology and sub-cellular distribution is provided in Table 3. The IMCL and mitochondrial content in both the SS and IMF sub-cellular regions were similar in lean and obese men (Figure 3, E–J). We undertook a novel analysis that characterizes the physical relationship between IMCL and mitochondria. There was no difference in the proportion of IMCL juxtaposed with mitochondria (i.e., the proportion of IMCL in contact with mitochondria) in both the SS (lean: 41.2±8.5% *vs.* obese: 39.7±4.6%) and IMF (lean: 40.9±15.2% *vs.* obese: 37.1±4.6%) regions between the two groups (Figure 4).

**Table 3:** Intramyocellular lipid and mitochondrial morphology.

	Lean		Obese		AdiposityEffect	TrainingEffect
	Pre-training	Post-training	Pre-training	Post-training		
**Intramyocellular lipid**						
IMCL size (µm^2^)						
Subsarcolemmal	0.21±0.02	0.14±0.02	0.25±0.04	0.17±0.01	*P* = 0.33	*P*≤0.01
Intermyofibrillar	0.15±0.02	0.19±0.04	0.15±0.02	0.23±0.03	*P* = 0.92	*P* = 0.09
No. of IMCL (#.µm^−2^)						
Subsarcolemmal	0.39±0.02	0.31±0.04	0.42±0.03	0.32±0.04	*P* = 0.36	*P*≤0.001
Intermyofibrillar	0.05±0.01	0.07±0.01	0.05±0.01	0.07±0.01	*P* = 0.83	*P*≤0.05
IMCL density (%)						
Subsarcolemmal	7.8±1.1	4.3±0.8	10.3±1.5	5.2±0.7	*P* = 0.20	*P*≤0.001
Intermyofibrillar	0.7±0.1	1.3±0.3	0.8±0.2	1.6±0.3	*P* = 0.44	*P*≤0.02
**Mitochondria**						
Mitochondria size (µm^2^)						
Subsarcolemmal	0.11±0.01	0.14±0.01	0.09±0.01	0.13±0.01	*P* = 0.19	*P*≤0.0001
Intermyofibrillar	0.09±0.01	0.13±0.01	0.09±0.01	0.13±0.01	*P* = 0.32	*P*≤0.001
No. of mitochondria (#.µm^−2^)						
Subsarcolemmal	1.43±0.16	1.53±0.14	1.31±0.19	1.31±0.12	*P* = 0.63	*P* = 0.80
Intermyofibrillar	0.40±0.04	0.45±0.04	0.37±0.05	0.45±0.06	*P* = 0.72	*P* = 0.20
Mitochondrial density (%)						
Subsarcolemmal	14.5±1.5	20.1±1.5	11.9±1.6	15.9±1.4	*P* = 0.27	*P*≤0.01
Intermyofibrillar	3.8±0.5	5.8±0.5	3.2±0.4	5.3±0.5	*P* = 0.35	*P*≤0.01
**IMCL juxtaposed mitochondria (%)**						
Subsarcolemmal	41.2±2.8	48.0±3.3	39.7±4.6	43.4±3.2	*P* = 0.79	*P* = 0.08
Intermyofibrillar	40.9±5.1	53.8±5.0	37.1±4.6	52.1±6.4	*P* = 0.59	*P*≤0.02

Data are presented as means ± SEM. IMCL, intramyocellular lipid.

#### Insulin signaling, glucose uptake and lipid metabolites

**Figure 5:**
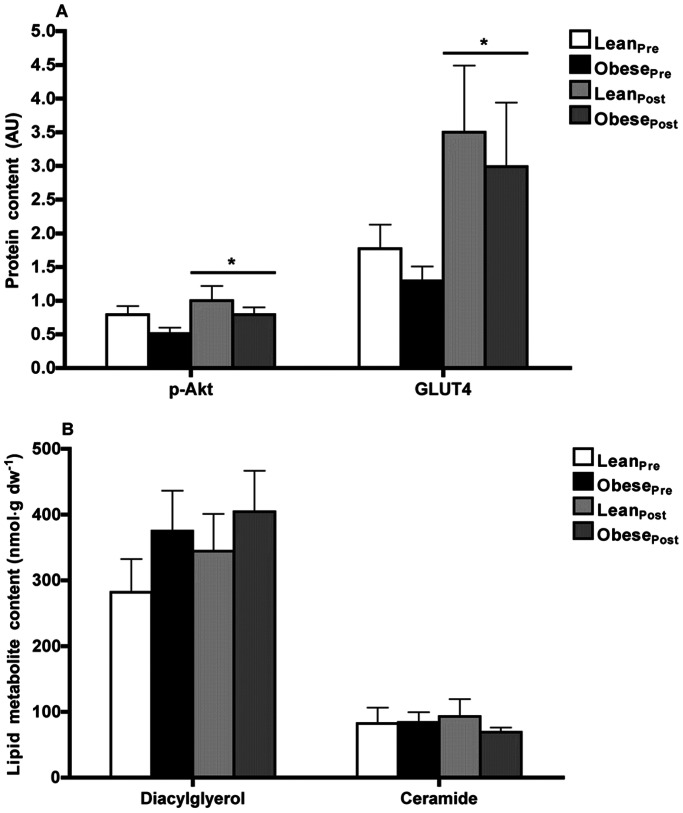
Insulin signaling and lipid metabolite data. (**A**) Akt phosphorylation at Ser473 residue and GLUT4 protein content assessed by Western blot and (**B**) diacylglycerol and ceramide lipid content in skeletal muscle of lean (*n* = 9) and obese (*n* = 9) men prior to and following 12-wk endurance training. (**A**) p-Akt, Akt phosphorylation at Ser473; GLUT4, glucose transporter 4. Results were normalized to β-actin protein content. * *P*≤0.03 pre- *vs.* post-training (main effect).

Basal Akt phosphorylation at Ser^473^ residue, a key step in insulin-stimulated glucose transport activity, and GLUT4 protein content were not different between the two groups (Figure 5A). We also examined intracellular fatty acid intermediates (DAG and ceramide) and did not observe any significant differences in these variables between the two groups (Figure 5B).

#### Correlations

HOMA-IR showed a positive correlation with BMI (*r* = 0.37; *P*≤0.03), waist circumference (*r* = 0.44; *P*≤0.01), and body fat percentage (*r* = 0.53; *P*≤0.01). Morphological measures of mitochondrial content (i.e., mitochondrial density), in the SS region (*r* = −0.34; *P* = 0.051) and IMF region (*r* = −0.29; *P* = 0.096) exhibited an inverse relationship with HOMA-IR (Table S1). A tendency for a positive relationship with HOMA-IR was found for SS IMCL size (*r* = 0.28; *P* = 0.119) and SS IMCL density (*r* = 0.25; *P* = 0.152) (Table S1). Regarding IMCL and mitochondria juxtaposition, HOMA-IR showed a tendency for an inverse relationship in both the SS region (*r* = −0.29; *P* = 0.101) and IMF region (*r* = −0.30; *P* = 0.086) (Table S2). HOMA-IR also showed a tendency for an inverse relationship with COXII (*r* = −0.32; *P* = 0.095) and COXIV (*r* = −0.35; *P* = 0.052) protein content (Table S3).

### Effect of Endurance Exercise Training

#### Subject characteristics

Twelve weeks of endurance exercise training increased aerobic capacity by 18% in lean men and by 15% in obese men (*P*≤0.001; Table 1). Waist circumference was reduced by 4% in lean men and by 3% in obese men (*P*≤0.001); whereas, body weight, BMI, fat mass, FFM, and body fat percentage were unchanged by training and remained markedly higher in the obese men (*P*≤0.02; Table 1). Endurance training increased FFA concentration (*P*≤0.03); whereas total cholesterol, triglyceride, HDL-cholesterol and LDL-cholesterol concentrations remained unchanged between groups (Table 2).

#### Oral glucose tolerance test and insulin resistance

Endurance training induced a significant reduction (19%) in the 2-h plasma glucose concentration (from 6.0±0.5 to 4.9±0.3, *P*≤0.01), and a 35% reduction in the 2-h plasma insulin concentration (from 49.7±10.6 to 32.4±3.8, *P* = 0.07). Endurance training had no effect on the plasma glucose concentration during the OGTT (AUC_glucose_) (Figure 1A). Endurance training had no effect on the plasma insulin concentration during the OGTT (AUC_insulin_), which remained significantly higher post-training in the obese group (Figure 1B). Endurance training tended to decrease HOMA-IR by 17% (2.17±0.19 *vs.* 2.60±0.30, post- *vs.* pre-training, respectively, *P* = 0.10; Figure 1C).

#### Biomarkers of mitochondrial content

Endurance training did not alter whole muscle PGC-1α protein content (Figure 2A) but did increase the protein content of CS (*P*≤0.04), COXII (*P*≤0.0001), and COXIV (*P*≤0.001) (Figure 2A) as well as the maximal activities of CS (*P*≤0.001), COX (*P*≤0.03), and SCHAD (*P*≤0.01) (Figure 2B). The effect of endurance training on muscle content of mtDNA was assessed for 8 of the 18 participants, those for whom sufficient pre- and post-training sample were available. Endurance training trended towards an increase in mtDNA copy number by 89% (1136±389 *vs*. 2782±1125, pre- *vs.* post-training, *P* = 0.10; Figure 2C).

#### Intramyocellular lipid and mitochondrial content

A summary of IMCL and mitochondrial morphology and sub-cellular distribution following endurance training is provided in Table 3. Lean and obese men had similar IMCL content in both the SS and IMF regions following endurance training (Figure 3). Endurance training decreased IMCL size in the SS region (−28%, *P*≤0.01), but tended to increase IMCL size in the IMF region (+40%, *P* = 0.09) (Figure 3E). Endurance training decreased the number of lipid droplets in the SS region (−22%, *P*≤0.001), but increased the number of lipid droplets in the IMF region (+49%, *P*≤0.05) (Figure 3F). Endurance training decreased IMCL density in the SS region (−43%, *P*≤0.001; Figure 3G), while it increased IMF IMCL density (+139%, *P*≤0.02; Figure 3G).

Lean and obese men had similar mitochondrial content in both the SS and IMF regions following endurance training (Figure 3). Endurance training increased mitochondria size in the SS region (*P*≤0.0001) and IMF region (*P*≤0.001) by a similar magnitude (40%) in both groups (Figure 3H). The total number of mitochondria in either the SS or IMF sub-cellular regions were unaffected by training (Figure 3I). Endurance training increased mitochondrial density in the IMF region (+83%, *P*≤0.01) to a greater degree than the SS region (+52%, *P*≤0.01) (IMF vs. SS, *P*≤0.03; Figure 3J). Endurance training increased the proportion of IMCL droplets juxtaposed with mitochondria by 19% in the SS region (*P* = 0.08), and by 55% in the IMF region (*P*≤0.02; Figure 4).

#### Insulin signaling, glucose uptake and lipid metabolites

Endurance training increased the phosphorylation of Akt^Ser473^ protein by 63% (from 0.65±0.08 to 0.90±0.12, *P*≤0.03) and resting whole muscle GLUT4 protein by 227% (from 1.52±0.21 to 3.26±0.67, *P*≤0.01; Figure 5A) but had no effect on either DAG or ceramide content (Figure 5B).

#### Correlations

Change in HOMA-IR (post-training vs. pre-training values) showed a positive correlation with a change in SS lipid droplet size (*r* = 0.55; *P* = 0.027) and a change in SS lipid droplet density (*r* = 0.50; *P* = 0.049) (Table S4). Additional correlation analyses are reported in Table S5 and Table S6.

## Discussion

In this study we have shown that several biomarkers of mitochondrial content were not differentially altered in skeletal muscle of obese men compared with healthy lean controls, despite the obese being insulin resistant. We also found that obese men did not have higher skeletal muscle IMCL, DAG or ceramide content than lean controls. It is important to note that our subjects were strictly matched for physical activity status, which we propose are vital measures that can confound the assessment of mitochondrial pathology associated with insulin resistance. The robust effect of moderate-intensity endurance exercise training stimulating mitochondrial capacity was clearly demonstrated but was not related to obesity or insulin sensitivity. The nature of the study design ensured that the potential confounding factors such as weight loss and/or dietary manipulation did not confound the interpretation of the true effects of endurance exercise training upon the variables measured in the current study. Our novel observation is that exercise training mediated a differential response on the localization, and magnitude of effect, upon both IMCL and mitochondrial cellular fractions. Finally, we found weak positive relationships between HOMA-IR values and SS lipid droplets, but a strong/moderate inverse relationship with mitochondrial density and IMCL/mitochondrial juxtaposition in both SS and IMF regions.

Our novel findings are supported by a growing body of evidence that indicates that insulin resistance is not strongly associated with mitochondrial capacity in muscle [37], [38], [39], [40], [41]. Discordance with studies supporting the etiological basis of mitochondrial dysfunction in insulin resistance may be explained by differences in study design (e.g., heterogeneity in physical fitness, co-morbidities associated with adiposity, age) and/or methodological differences in assessing mitochondrial function. In contrast to other studies [10], [11], where a large difference in demographics existed between the groups, participants in our study were appropriately age- and physical activity-matched sedentary, non-diabetic, lean and obese men. Considering that individuals with obesity, insulin resistance and T2D are generally physically inactive, the impairments in oxidative metabolism might simply be attributed to their sedentary lifestyle and thus the previous associations between muscle mitochondrial function and insulin resistance may be confounded [41], [42], [43]. This conclusion is supported by a more recent genome-wide RNA expression analyses that found that the skeletal muscle transcriptome in T2D was indistinguishable from that of normal glucose tolerant subjects when subjects were well-matched for fitness [44]. Additionally, insulin resistant obese men in the current study experienced robust increases in mitochondrial content and oxidative function to the same extent as lean controls in response to endurance training. Such increases in mitochondrial content/function were observed in the absence of changes in body composition. Together these observations indicate that physical activity is the chief factor modulating overall skeletal muscle mitochondrial capacity, whereas reduced skeletal muscle mitochondrial content/function only partially mediates insulin resistance.

The hallmark of the theory linking insulin resistance to mitochondrial dysfunction is the accumulation of IMCL whose altered metabolism impairs insulin signaling [13], [22], [25]. This notion is challenged by the “athlete’s paradox” where an increase in IMCL content in endurance-trained athletes is coupled with increased mitochondrial content and heightened insulin sensitivity [15], [26], [29]. To gain an insight into the proposed IMCL accumulation phenomenon, we carried out an in-depth analysis of skeletal muscle IMCL accumulation in this study. We utilized TEM to specifically assess IMCL content in both SS and IMF regions and found no difference in skeletal muscle IMCL content between lean and obese men. This result was inconsistent with the work by Nielsen and colleagues in which a higher level of SS lipids but not IMF lipids was reported in obese type 2 diabetic patients compared with obese non-diabetics and endurance-trained subjects [27]. However, Nielson *et al.* did not report an analysis between obese non-diabetics and fitness-matched lean subjects, which precludes comparisons with the present study, in which all subjects were non-diabetic and matched for physical activity status and fitness. Similarly, we did not observe a difference in mitochondrial subpopulations in insulin-resistant obese *vs.* lean men, contrasting with a former report of a depletion of SS mitochondria [3]. A shortcoming of the study by Ritov *et al.* [3] was that aerobic capacity was not quantified in the study participants, nor was age controlled for. Furthermore, mitochondria were not directly quantified, instead the thickness of the layer of SS mitochondria was used as a marker of mitochondrial mass [3]. The SS space thickness may not be the best assessment of mitochondrial content, since the SS space can be influenced by lipid droplets and nuclei occupying it. Here, we used more rigorous methodology to measure mitochondrial content. Furthermore, in comparison with Ritov *et al.*, our obese men were matched with lean controls thereby diminishing the confounding effects of differences in physical activity habits between groups. Each or all of these factors could have contributed to the difference in findings between studies.

The effect of endurance exercise training on IMCL content in skeletal muscle is equivocal. While endurance exercise has previously been shown to lower IMCL content in T2D [45], [46], [47], we and others have shown that in non-diabetic untrained subjects, endurance training elevates IMCL content [29], [48], [49], presumably as an adaptation to maximize surface area and total muscle IMCL content for lipolysis during exercise. We report here the novel findings that IMCL content is differentially regulated at the sub-cellular level in myofibers in response to endurance training. In both lean and obese men, endurance training mediated a significant decrease in IMCL size, number and density in the SS region, whereas the opposite effect was observed in IMCL size, number and density in the IMF region. We additionally demonstrated that the proportion of IMCL droplets juxtaposed with mitochondria increased in both lean and obese groups post-training in both sub-cellular regions, potentially contributing to more efficient substrate oxidation as noted by a concomitant increase in β-oxidation following endurance training in both groups. This novel finding extends some of our previous analyses conducted in non-obese populations [29], [50]. Perhaps greater efficiency of substrate oxidation through tighter coupling of IMCL droplets and mitochondria is more indicative of whole-body insulin sensitivity; as such, we found moderate inverse associations between HOMA-IR and IMCL/mitochondria juxtaposition in both the SS and IMF regions.

Another new finding from this study is that skeletal muscle mitochondria respond to training even in obese men with insulin resistance. We have shown that mitochondrial size and density, but not number, increase in both the SS and IMF regions; and this effect is independent of lean and obese groups. This finding confirms our previous report of increases in total mitochondrial volume density due to an increase in the size of the mitochondrial reticulum and not the number of fragments in response to endurance exercise [29]. In comparison with IMF mitochondria, SS mitochondria respond to a greater degree, or earlier in time, to training [27], [48]. In the current study, the training-mediated increase in mitochondrial density in the IMF (+83%) was greater than the increase in SS mitochondrial density (+52%); opposite to the pattern previously observed [27], [48]. The differences in study population, duration of endurance exercise training, type I and II muscle fiber distribution, and the sub-cellular image sampling methods may partially explain the observed difference.

More active lipid metabolites (DAG and ceramide) have been implicated in impaired insulin signaling [20], [51], [52], [53]. Itani et al. found that insulin sensitivity decreased after increasing plasma NEFA with a lipid infusion during a euglycemic–hyperinsulinemic clamp, and this occurred in parallel with an increase in intramuscular DAG, but not ceramide [20]. Straczkowski et al. [53] showed that lipid infusion reduced insulin sensitivity, and this coincided with increased muscle ceramide content. Lastly, Bruce et al. found that glucose tolerance was increased and muscle ceramide levels were decreased after 8 weeks of training [54]. In the present study, we did not find an effect of endurance exercise training on DAG or ceramide content (despite an increase in FFAs), nor differences between these lipid intermediates in lean and obese men at baseline, supporting recent human [55] and rodent [56] data. Additionally, Serlie et al. found lean and overweight individuals to have similar muscle ceramide concentrations [57]. It is not easy to reconcile these findings, but it can be speculated that a threshold of obesity may exist, above which muscle ceramide accumulates and influences insulin signaling and thus insulin sensitivity. Furthermore, we cannot exclude the possibility that muscle long chain fatty acyl-CoA may have influenced insulin sensitivity. Given that robust changes in mitochondrial and β-oxidation capacity occurred without alterations in DAG or ceramide muscle content in the present study further adds to the complexity in understanding the link between IMCL-mitochondria and glucose homeostasis.

We did not find any differences in phosphorylation of Akt^Ser473^, a key downstream step for the activation of glucose transport, and GLUT4 protein contents between lean and obese men. Although previous investigations have reported reduced Akt activity [52], [58], it is important to note that these studies were conducted in cultured muscle cells from severely obese women under insulin stimulated conditions [58] or obese men and women during euglycemic-hyperinsulinemic clamp settings [52], and it is not clear whether physical activity and/or physical fitness were strictly controlled for; consequently, their findings may not be representative of obese individuals with less severe pathology. In a previous study, Hood et al. reported reduced skeletal muscle Akt phosphorylation in the basal state in response to endurance training (high-intensity interval training) in healthy men and women [59]. Liu et al. have recently hypothesized that basal Akt activation in skeletal muscle is linked to reduced mitochondrial content and insulin resistance [60]. Akt has been shown to directly phosphorylate PGC-1α, leading to its inhibition and degradation [61]. In response to a high-fat diet, mice become insulin resistant concomitant with an increase in basal Akt activation, a reduction in PGC-1α protein, and a decrease in mitochondrial content in skeletal muscle [60]. In response to 12 weeks of endurance exercise training, we observed a trend in decreasing insulin resistance (HOMA-IR), an increase in basal Akt activation, and an increase in mitochondrial content in skeletal muscle. Although these findings conflict with the aforementioned studies suggesting a link between elevated basal Akt activation and insulin resistance, our study underscores the complexity of the issue linking insulin signaling pathways in obesity and exercise. The absence of insulin signaling impairments coincide with the lack of higher IMCL content and no differences in DAG or ceramide in the skeletal muscle of insulin resistant obese men, which in turn are consistent with our measurements showing uncompromised mitochondrial function/β-oxidation and mitochondrial morphology. Together, these results suggest that when confounding factors such as age and physical activity status are controlled for; insulin resistance is only partially explained by skeletal muscle mitochondrial content and IMCL content or distribution, but not DAG or ceramides. This conclusion is further supported by our previous work in older adults where we have shown that mitochondrial function was not just influenced by age *per se*, but was strongly modulated by the physical activity status of the participants [28], [29].

We did not observe a clear effect of endurance training on whole-body insulin resistance. It is important to note that this observation was made in the absence of body composition changes (i.e., weight loss); and a ∼20% increase in aerobic capacity. Our observation is in accordance with our previous study in which three months of moderate-intensity cycling did not improve insulin resistance in lean and obese women [30]. Of note, women in the latter study did not lose body weight or alter body composition after exercise training. These results are consistent with those reporting only modest or no improvements in insulin sensitivity with exercise training in middle-aged or older men and women [62] or when the effects of weight loss [63] and the last exercise session [64] are accounted for.

It must be acknowledged that the present findings are based on a small sample of inactive adult men. As such, certain results may be attributed to false-negatives due to the lack of statistical power. For example, the present study had only 56% power to detect a statistically significant difference in HOMA-IR in response to endurance exercise training. In order to have potentially observed a statistical reduction in HOMA-IR, given the variability in our study population, approximately 88 adult men would have had to be enrolled. Despite this, however, we were able to detect significant changes in many muscle metabolic parameters after 12 weeks of endurance training. As this study was conducted in men only, and given that sex differences exist in the relationship between IMCL and obesity or insulin resistance, our study limits any direct comparisons to studies reporting data in both men and women. On the basis of our encouraging findings for the efficacy of moderate-intensity endurance exercise training for improving markers of metabolic health, future studies should include larger sample sizes, including both men and women, and a type 2 diabetic group to directly assess the potential health-promoting adaptations to this type of training. Future studies should also use more direct measures of muscle insulin sensitivity, glycemic control, and mitochondrial function because the assessment of insulin resistance using HOMA-IR and the assessment of mitochondrial function using mitochondrial proteins and enzyme activities of mitochondrial complexes in the present study is limited by the fact that it was based with a single fasting blood sample and frozen muscle samples, respectively. Muscle glucose uptake is primarily regulated by insulin signaling to GLUT4 translocation from intracellular pools to the sarcolemma. We assessed total GLUT4 protein content in resting muscle biopsy samples in this study and as such cannot determine whether endurance exercise training had an influence on GLUT4 trafficking. However, an increase in total GLUT4 is a relatively rapid response and seems to be important in mediating some of the increase in muscle glucose uptake and insulin sensitivity after exercise [65], [66], [67]. Finally, quantification of IMCL and mitochondria in humans is further complicated by a heterogeneous myocyte or fiber composition. Type I fibers have been reported to be less abundant in obese and/or type 2 diabetic patients [68], but it is unclear if these differences persist when controlled for physical activity/fitness. Although we have substantiated our TEM data with additional biochemical markers of mitochondrial function and insulin resistance, fiber type composition was not controlled for, hence, additional studies are needed to link IMCL, fiber type, oxidative capacity, and activation of specific insulin-signaling proteins.

In conclusion, our results do not primarily support the notion of impaired skeletal muscle mitochondrial function or IMCL/DAG/ceramide accumulation *per se* being linked to insulin resistance in obese men. Previous associations may in part be due to other confounders, such as age, co-morbidities and physical activity. Additionally, this study illustrates that moderate-intensity endurance exercise training, in the absence of weight loss, evokes favorable mitochondrial adaptations in both lean and insulin-resistant obese men to a similar extent and differentially regulates intracellular subpopulations of both IMCL and mitochondria. Future studies are warranted to further ascertain the molecular deficits that lead to insulin resistance in the absence of the confounding variables of age, physical activity level and diet. We believe that in light of our current findings and previous contradictory reports where confounding variables were not strictly controlled for, it is imperative to test if insulin resistance plays a causal role in promoting mitochondrial dysfunction systemically in obese subjects and patients with type 2 diabetes. Additionally, it will be of great interest to decipher if insulin resistance-mediated hormonal dysfunction in obesity modulates the effects of physical activity and promotes secondary pathology, such as skeletal muscle mitochondrial dysfunction.

## Supporting Information

Table S1 **Pearson Correlation Analyses, HOMA-IR vs. Mitochondria and IMCL Morphology Features.**
(DOCX)

Table S2 **Pearson Correlation Analyses, HOMA-IR vs. IMCL and Mitochondria Juxtaposition.**
(DOCX)

Table S3 **Pearson Correlation Analyses, HOMA-IR vs. Mitochondria Biochemical Features and Lipid Intermediates.**
(DOCX)

Table S4 **Pearson Correlation Analyses, Change in HOMA-IR vs. Change in Mitochondria and IMCL Morphology Features.**
(DOCX)

Table S5 **Pearson Correlation Analyses, Change in HOMA-IR vs. Change in IMCL and Mitochondria Juxtaposition.**
(DOCX)

Table S6 **Pearson Correlation Analyses, Change in HOMA-IR vs. Change in Mitochondria Biochemical Features and Lipid Intermediates.**
(DOCX)
