# Supplementary material for: Markers of Skeletal Muscle Mitochondrial Function and Lipid Accumulation Are Moderately Associated with the Homeostasis Model Assessment Index of Insulin Resistance in Obese Men
Source: PLoS One. 2013 Jun 12;8(6):e66322. doi: 10.1371/journal.pone.0066322 (PMC3680409; doi:10.1371/journal.pone.0066322)
Supplement: Table S1 — Pearson Correlation Analyses, HOMA-IR vs. Mitochondria and IMCL Morphology Features. (DOCX) [file pone.0066322.s001.docx]

**Table S1**: Pearson Correlation Analyses, HOMA-IR vs. Mitochondria and IMCL Morphology Features

|  | Mitochondria | | | | | | Intramyocellular Lipid | | | | | |
| --- | --- | --- | --- | --- | --- | --- | --- | --- | --- | --- | --- | --- |
|  | Size | | Number | | Density | | Size | | Number | | Density | |
|  | SS | IMF | SS | IMF | SS | IMF | SS | IMF | SS | IMF | SS | IMF |
| **HOMA-IR** | r = -0.23 | r = -0.13 | r = -0.18 | r = -0.21 | r = -0.34 | r = -0.29 | r = 0.28 | r = -0.06 | r = 0.03 | r = -0.12 | r = 0.25 | r = -0.06 |
| ***P* Value** | 0.189 | 0.459 | 0.305 | 0.230 | 0.051 | 0.096 | 0.119 | 0.739 | 0.856 | 0.509 | 0.152 | 0.732 |

HOMA-IR, homeostasis model assessment index of insulin resistance; IMF, intermyofibrillar; SS, subsarcolemmal
