# Supplementary material for: Markers of Skeletal Muscle Mitochondrial Function and Lipid Accumulation Are Moderately Associated with the Homeostasis Model Assessment Index of Insulin Resistance in Obese Men
Source: PLoS One. 2013 Jun 12;8(6):e66322. doi: 10.1371/journal.pone.0066322 (PMC3680409; doi:10.1371/journal.pone.0066322)
Supplement: Table S3 — Pearson Correlation Analyses, HOMA-IR vs. Mitochondria Biochemical Features and Lipid Intermediates. (DOCX) [file pone.0066322.s003.docx]

**Table S3:** Pearson Correlation Analyses, HOMA-IR vs. Mitochondria Biochemical Features and Lipid Intermediates

|  | Protein Content | | | | Enzyme Activity | | mtDNA | DAG | Ceramide |
| --- | --- | --- | --- | --- | --- | --- | --- | --- | --- |
|  | CS | COXIV | COXII | PGC-1α | CS | COX |  |  |  |
| **HOMA-IR** | r = -0.19 | r = -0.35 | r = -0.32 | r = -0.11 | r = -0.04 | r = 0.06 | r = -0.36 | r = -0.01 | r = -0.08 |
| ***P* Value** | 0.272 | 0.052 | 0.095 | 0.595 | 0.841 | 0.753 | 0.159 | 0.952 | 0.658 |

COX, cytochrome *c* oxidase - subunit II/IV; CS, citrate synthase, DAG, diacylglycerol; HOMA-IR, homeostasis model assessment index of insulin resistance; mtDNA, mitochondrial DNA; PGC-1 α, peroxisome proliferator-activated receptor-γ coactivator-1α
