# Supplementary material for: Markers of Skeletal Muscle Mitochondrial Function and Lipid Accumulation Are Moderately Associated with the Homeostasis Model Assessment Index of Insulin Resistance in Obese Men
Source: PLoS One. 2013 Jun 12;8(6):e66322. doi: 10.1371/journal.pone.0066322 (PMC3680409; doi:10.1371/journal.pone.0066322)
Supplement: Table S4 — Pearson Correlation Analyses, Change in HOMA-IR vs. Change in Mitochondria and IMCL Morphology Features. (DOCX) [file pone.0066322.s004.docx]

**Table S4:** Pearson Correlation Analyses, Change in HOMA-IR vs. Change in Mitochondria and IMCL Morphology Features

|  | Mitochondria | | | | | | Intramyocellular Lipid | | | | | |
| --- | --- | --- | --- | --- | --- | --- | --- | --- | --- | --- | --- | --- |
|  | Δ Size | | Δ Number | | Δ Density | | Δ Size | | Δ Number | | Δ Density | |
|  | SS | IMF | SS | IMF | SS | IMF | SS | IMF | SS | IMF | SS | IMF |
| **ΔHOMA-IR** | r = 0.10 | r = 0.16 | r = -0.04 | r = -0.04 | r = 0.03 | r = 0.06 | r = 0.55 | r = 0.06 | r = -0.42 | r = -0.37 | r = 0.50 | r = -0.15 |
| ***P* Value** | 0.707 | 0.543 | 0.880 | 0.889 | 0.923 | 0.815 | 0.027 | 0.823 | 0.102 | 0.157 | 0.049 | 0.569 |

HOMA-IR, homeostasis model assessment index of insulin resistance; IMF, intermyofibrillar; SS, subsarcolemmal
