# Supplementary material for: Markers of Skeletal Muscle Mitochondrial Function and Lipid Accumulation Are Moderately Associated with the Homeostasis Model Assessment Index of Insulin Resistance in Obese Men
Source: PLoS One. 2013 Jun 12;8(6):e66322. doi: 10.1371/journal.pone.0066322 (PMC3680409; doi:10.1371/journal.pone.0066322)
Supplement: Table S5 — Pearson Correlation Analyses, Change in HOMA-IR vs. Change in IMCL and Mitochondria Juxtaposition. (DOCX) [file pone.0066322.s005.docx]

**Table S5:** Pearson Correlation Analyses, Change in HOMA-IR vs. Change in IMCL and Mitochondria Juxtaposition

|  | Δ IMCL Juxtaposed to Mitochondria | |
| --- | --- | --- |
|  | SS | IMF |
| **ΔHOMA-IR** | r = 0.05 | r = 0.10 |
| ***P* Value** | 0.861 | 0.703 |

HOMA-IR, homeostasis model assessment index of insulin resistance; IMCL, intramyocellular lipid; IMF, intermyofibrillar; SS, subsarcolemmal
