# Supplementary material for: Markers of Skeletal Muscle Mitochondrial Function and Lipid Accumulation Are Moderately Associated with the Homeostasis Model Assessment Index of Insulin Resistance in Obese Men
Source: PLoS One. 2013 Jun 12;8(6):e66322. doi: 10.1371/journal.pone.0066322 (PMC3680409; doi:10.1371/journal.pone.0066322)
Supplement: Table S6 — Pearson Correlation Analyses, Change in HOMA-IR vs. Change in Mitochondria Biochemical Features and Lipid Intermediates. (DOCX) [file pone.0066322.s006.docx]

**Table S6:** Pearson Correlation Analyses, Change in HOMA-IR vs. Change in Mitochondria Biochemical Features and Lipid Intermediates

|  | Protein Content | | | | Enzyme Activity | | Δ mtDNA | Δ DAG | Δ Ceramide |
| --- | --- | --- | --- | --- | --- | --- | --- | --- | --- |
|  | Δ CS | Δ COXIV | Δ COXII | Δ PGC-1α | Δ CS | Δ COX |  |  |  |
| **ΔHOMA-IR** | r = -0.38 | r = -0.28 | r = -0.12 | r = 0.33 | r = -0.06 | r = 0.05 | r = -0.16 | r = 0.04 | r = -0.16 |
| ***P* Value** | 0.145 | 0.354 | 0.692 | 0.344 | 0.812 | 0.840 | 0.728 | 0.889 | 0.574 |

COX, cytochrome *c* oxidase - subunit II/IV; CS, citrate synthase, DAG, diacylglycerol; HOMA-IR, homeostasis model assessment index of insulin resistance; mtDNA, mitochondrial DNA; PGC-1 α, peroxisome proliferator-activated receptor-γ coactivator-1α
